# Global phylogeography and genetic diversity of the long-finned pilot whale *Globicephala melas*, with new data from the southeastern Pacific

**DOI:** 10.1038/s41598-020-58532-3

**Published:** 2020-02-04

**Authors:** Sebastián Kraft, MJosé Pérez-Álvarez, Carlos Olavarría, Elie Poulin

**Affiliations:** 1 Instituto de Ecología y Biodiversidad, Laboratorio de Ecología Molecular, Departamento de Ciencias Ecológicas, Facultad de Ciencias, Universidad de Chile, Santiago, Chile; 2 Centro de Investigación Eutropia, Santiago, Chile; 3 Escuela de Medicina Veterinaria, Facultad de Ciencias, Universidad Mayor, Santiago, Chile; 4 Centro de Estudios Avanzados en Zonas Áridas (CEAZA), La Serena, Chile

**Keywords:** Biogeography, Ecological genetics

## Abstract

The matrilineal long-finned pilot whale presents an antitropical distribution and is divided into two subspecies, one in the temperate seas of the Southern Hemisphere and the other restricted to the North Atlantic and Mediterranean. Until now, population genetic and phylogeographic studies have included localities of most of its Northern Hemisphere distribution, while only the southwestern Pacific has been sampled in the Southern Hemisphere. We add new genetic data from the southeastern Pacific to the published sequences. Low mitochondrial and nuclear diversity was encountered in this new area, as previously reported for other localities. Four haplotypes were found with only one new for the species. Fifteen haplotypes were detected in the global dataset, underlining the species’ low diversity. As previously reported, the subspecies shared two haplotypes and presented a strong phylogeographic structure. The extant distribution of this species has been related to dispersal events during the Last Glacial Maximum. Using the genetic data and Approximate Bayesian Calculations, this study supports this historical biogeographic scenario. From a taxonomic perspective, even if genetic analyses do not support the subspecies category, this study endorses the incipient divergence process between hemispheres, thus maintaining their status and addressing them as Demographically Independent Populations is recommended.

## Introduction

Cetaceans have a diverse -and many times contrasting- array of geographic distributions. Some of the widest ranging species include the cosmopolitan sperm whale *Physeter macrocephalus*^1^ and the orca *Orcinus orca*^2^. Other species, especially small-sized odontocetes, generally present coastal and more restricted distributions, like the extreme case of the vaquita, *Phocoena sinus*^3^.

Some cetaceans have a particular distribution pattern known as disjunct or antitropical, in which the taxon is present at high latitudes in both hemispheres while being absent from lower latitudes^4^. Examples include the mysticete genus *Eubalaena*, with *E. borealis* inhabiting the North Pacific, *E. japonica* the North Atlantic and their austral equivalent in the Southern Hemisphere, *E. australis*^5^. The phocoenid species pair *Phocoena phocoena* and *P. spinipinnis* is another example, found in the Northern Hemisphere and the coasts of South America, respectively^6^. Among delphinids, the two *Lissodelphis* species also present this antitropical distribution pattern; *L. peronii* is present around the Southern Hemisphere, while *L. borealis* is found in the North Pacific^7^. A similar antitropical distribution is presented by the two subspecies of long-finned pilot whales *Globicephala melas*, where *G. m. edwardii* inhabits the temperate to subpolar waters of the Southern Hemisphere, while *G. m. melas* is restricted to the North Atlantic^8^. Extinct populations of long-finned pilot whales have been reported in the North Pacific, from Japan^9^ and Alaska^10^, dating back 8 000–12 000 years and 2 500–3 500 years, respectively^9,10^.

Pilot whales are a highly social matrilineal odontocete, thought to be among the most gregarious cetaceans. The species is known to form groups with a mean size around tens of individuals^11^ that can increase to hundreds through aggregations^12,13^. These groups are structurally based on strong matrilineal associations^6,8^ of closely related females and their descendants^14^. Pilot whales are among the most common cetaceans involved in mass strandings^6,8,13^. Southern Hemisphere long-finned pilot whales were originally described as a distinct species, *G. leucosagmaphora*^15^, but eventually ranked as one of the two subspecies of *G. melas*^16^, based on observations of the coloration pattern and morphology^16,17^. Sergeant (1962) stated that he found only minor differences in coloration and none in external morphology between specimens from the two hemispheres, albeit being in agreement with the subspecies classification proposed by Davies (1960)^16^. The author also expressed the need of including samples from additional localities, in order to assess the variation of the colour pattern in the subspecies, as also recommended by other authors^18^. A more recent study found differences in skull morphometry of North and South Atlantic specimens^19^, but no studies were found that account for geographic variation within each area. Little genetic evidence exists to support their classification status using mitochondrial DNA. Oremus *et al*., (2009) performed the first inter-hemisphere comparison of the taxa, between the southwestern Pacific and the North Atlantic^20^. The authors also stated that the two subspecies do not qualify as Evolutionary Significant Units (ESU) according to the reciprocal monophyly criterion of Moritz (1994)^21^, only reporting restrictions to gene flow among the areas of distribution mainly based on differences in the frequency of shared haplotypes. In the study of Oremus *et al*., (2009), preliminary genetic data support a biogeographic scenario previously proposed by Davies (1960)^22^. A colonization event from south to north would have taken place through a founder effect, followed by demographic population growth. Additional genetic research on pilot whales has been conducted mostly at the population level in the North Atlantic and Mediterranean Sea^23–25^, however, an integration of this available information is missing, together with the sampling of other areas such as the southeastern Pacific.

In this study, we include new genetic data and results from samples collected in two mass standings in southern Chile, in order to improve the global phylogeographic overview of long-finned pilot whales. We also evaluate the historical biogeographic processes that originated the extant antitropical distribution of this species and discuss its taxonomic status.

## Results

### Southeastern pacific sampling and genetic diversity

In total, 90 mtDNA sequences were obtained, defining four haplotypes. One haplotype was previously unreported (hereon referred to as haplotype R2). Haplotype and nucleotide diversities were low: Hd = 0.62 and π = 0.23% (Table 1).

**Table 1:** Summary of number of haplotypes and genetic indices for each included locality and per subspecies (abbreviations are detailed in the methods section). The first row indicates the names of each haplotype according to the original authors.

	S + R	P + U	Q + Y	R2	T	O	V	O2	W	Z	E + G	60	62	X	D	Total	h	S	Hd	*π*%	Π
TAS	51	32	72													215	6	4	0.783	0.394	1.356
NZ	6	333	3		1	14	1									358	6	5	0.133	0.040	0.136
CL	31	45	12	2												90	4	4	0.620	0.230	0.791
NWA	73	1												4		78	3	2	0.123	0.064	0.220
FI	55	17														72	2	1	0.365	0.100	0.365
UK	34	1									4					39	3	2	0.235	0.070	0.240
IB	39										1	1	1			42	4	3	0.139	0.055	0.188
NEA	20										1					21	2	1	0.095	0.027	0.095
GIB	44														20	64	2	1	0.436	0.126	0.436
MED	12														21	33	2	1	0.477	0.138	0.477
*G. m. edwardii*	88	410	87	2	1	14	1	14	15	31	6					663	10	9	0.580	0.233	0.802
*G. m. melas*	277	19										1	1	4	41	349	8	5	0.353	0.117	0.404
Total	365	429	87	2	1	14	1	14	15	31	6	1	1	4	41	1012	15	12	0.680	0.264	0.909

Numbers 60 and 62 represent the two last digits of the GenBank codes provided by Miralles et al., (2016) for identification. Number of haplotypes (h), haplotype diversity (Hd), pairwise differences between sequences (Π) and nucleotide diversity (π) are also detailed.

Microsatellites were successfully genotyped in n = 44 samples (32 from Isla Clemente and 12 from Isla Navarino), across fourteen polymorphic loci. DlrFCB6, and GT51 presented an excess of homozygotes, but were not used in the comparisons among hemispheres. Regional diversity values of usable loci were *H*_*o*_ = 0.655; *H*_*e*_ = 0.700 and nA = 7.5 (Table 2).

**Table 2:** Comparisons among shared microsatellite loci from three studies including Tasmania and New Zealand^27^, Chile (this study) and the North Atlantic^28^. Dashes indicate either unreported information or no locus amplification.

Locus	Tasmania & New Zealand				Chile				North Atlantic			
	n	Alleles	*H*_*o*_	*H*_*e*_	n	Alleles	*H*_*o*_	*H*_*e*_	n	Alleles	*H*_*o*_	H_e_
409/470	262	10	0.844	0.825	—	—	—	—	529	9	—	0.567
415/416	242	9	0.798	0.801	—	—	—	—	529	5	—	0.567
464/465	122	9	0.648	0.681	44	9	0.676	0.578	529	6	—	0.670
DlrFCB1	264	15	0.777	0.774	44	8	0.682	0.760	—	—	—	—
DlrFCB6	256	7	0.672	0.693	44	7	0.476	0.716	—	—	—	—
EV1	262	14	0.756	0.773	44	10	0.756	0.739	—	—	—	—
EV37	263	10	0.814	0.775	44	15	0.633	0.858	529	6	—	0.748
EV94	255	7	0.620	0.686	—	—	—	—	529	7	—	0.772
GT23	263	5	0.468	0.439	44	4	0.341	0.567	—	—	—	—
GT39	122	10	0.787	0.822	44	5	0.636	0.512	—	—	—	—
GT51	253	3	0.300	0.308	44	4	0.318	0.492	—	—	—	—
GT575	254	11	0.827	0.836	44	7	0.841	0.828	—	—	—	—
MK5	244	6	0.623	0.658	44	7	0.682	0.670	—	—	—	—
MK9	120	4	0.625	0.618	44	4	0.591	0.613	—	—	—	—
PPHO131	248	10	0.734	0.745	44	6	0.714	0.696	—	—	—	—

### Global diversity analysis

After adding previously published sequences from other oceanic basins^20,23–26^, a total of 15 haplotypes were obtained after the elimination of the site that was generating phylogeographic noise in the total 1012 sequences. This led to the combination of four previously described haplotypes in the following pairs: S with R, P with U, Q with Y, and E with G (Table 1). Nine of the twelve haplotypes reported by Miralles *et al*., (2016), originally of a consensus fragment size of 703 bp, were also merged into haplotype S + R.

### Global and local haplotype networks

**Figure 1:**
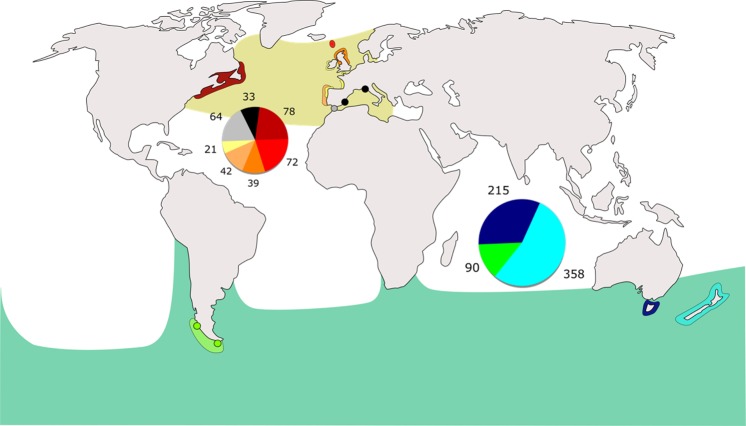
Origin of the 1012 sequences included in the study: Chile (green), Tasmania (dark blue), New Zealand (light blue), northwestern Atlantic (dark red), Faroe Islands (red), United Kingdom (orange), Iberian Peninsula (light orange), northeastern Atlantic (yellow), Strait of Gibraltar (grey), Mediterranean (black). Pie charts indicate the number of sequences contained in each locality. Ocean areas in yellow represent the extant distribution of *G. m. melas* and in green of *G. m. edwardii*. Map adapted from SVG SILH (https://svgsilh.com/image/306338.html), released as public domain under Creative Commons CC0 1.0. Figure generated by S.K. in Inkscape 0.92.4 (https://www.inkscape.org).

Adding previously published sequences from other oceanic basins, we detected that (1) two haplotypes were shared between *G. m. edwardii* (SP) and *G. m. melas* (NA and MED), and (2) one haplotype was shared by all SP localities but was absent from NA and MED (Table 1, Fig. 1).

**Figure 2:**
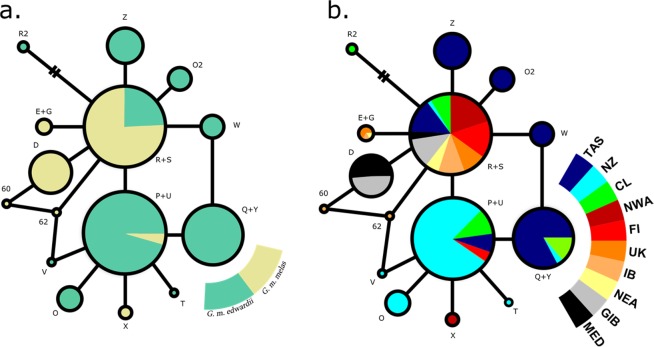
Haplotype networks of (**a**) the global data coloured by subspecies: *G. m. melas* in yellow and *G. m. edwardii* in green; and (**b**) coloured by locality: Tasmania (TAS), New Zealand (NZ), Chile (CL), northwestern Atlantic (NWA), Faroe Islands (FI), United Kingdom (UK), Iberian Peninsula (IB), northeastern Atlantic (NEA), Strait of Gibraltar (GIB) and the Mediterranean Sea (MED). Smallest circle size indicates a frequency of one sequence. Detailed haplotype frequencies can be found in Table 1.

Haplotypes P + U and S + R were the only ones detected within the distribution range of both *G. melas* subspecies (Table 1, Fig. 2a): haplotype P + U is present in the SP and NA, while S + R was found in all three basins (SP, NA and MED), and was pivotal in the development of local diversity. Haplotype Q + Y was shared by all SP localities, but absent from NA and MED. The remaining 12 haplotypes represent *in situ* diversification within each corresponding basin: eight for SP, four for NA and one for MED. The haplotypes found in the Mediterranean area are represented either by S + R or by D, which derives directly from it. The same occurs with half (2) of haplotypes encountered in the NA and half (4) of those exclusive to the SP.

In the haplotype network by locality, haplotype S + R is present in all ten studied areas and originates much of the local diversity within them (Table 1, Fig. 2b). Haplotype P + U plays a similar, but downscaled role, in the South Pacific; it originates half (4) of the diversity that is exclusive to this area. This haplotype is also much more abundant in the localities corresponding to *G. m. edwardii* than in the range of *G. m. melas*, where it was detected in lower numbers in three localities (17 samples from FI, one from NWA and one from UK). One of the haplotypes that originates from P + U, haplotype Q + Y, is exclusive to the South Pacific and is present in all three localities. Ten of the total fifteen haplotypes (67%) were exclusively present in one of the following five localities: TAS (3), NZ (3), CL (1), IB (2) and NWA (1). In the South Pacific, 70% of haplotypes were private to one of the three localities; it was the only basin with at least one private haplotype in each. Three private haplotypes were encountered in the North Atlantic: one in the NWA and two in the IB.

### Global and local genetic structure

Significant genetic and phylogeographic structure was also detected between the subspecies, *i.e*. South Pacific with North Atlantic and Mediterranean (*F*_*ST*_ = 0.439, *φ*_*ST*_ = 0.464, both P < 0.000004). The Snn test for genetic differentiation was statistically significant among the two disjoint distribution areas of the two subspecies (*i.e. G. m. edwardii* and *G. m. melas*; Snn = 0.830, P < 0.001). Subsequently, samples were organized in this same way for the AMOVA. Similar structure results were obtained grouping the samples by the 10 worldwide localities. Average *φ*_*ST*_ values were highest within the SP (avg. *φ*_*ST*_ = 0.283), lowest in the NA (avg. *φ*_*ST*_ = 0.06), and intermediate among the two Mediterranean localities (avg. *φ*_*ST*_ = 0.174) (Table 3). All *φ*_*ST*_ comparisons were statistically significant within the South Pacific. In the North Atlantic, only the Faroe Islands showed a statistically significant phylogeographic structure with all other localities, while NWA did with FI and UK. All comparisons among localities from different basins showed statistically significant differences. The AMOVA showed significant differentiation among the two distribution ranges, with greater variation among groups than within them (Table 4).

**Table 3:** Genetic (*F*_*ST*_) and phylogeographic structure (*φ*_*ST*_) values of the comparisons among the ten worldwide sampling localities. Structure values are beneath each diagonal and P-values are above them.

^F^ST	NWA	FI	UK	IB	NEA	GIB	MED	TAS	NZ	CL
NWA	—	0	0.057	0.394	0.616	0	0	0	0	0
FI	0.136	—	0.019	0.001	0.034	0	0	0	0	0
UK	0.034	0.077	—	0.270	0.559	0.001	0	0	0	0
IB	−0.001	0.115	0.006	—	0.999	0	0	0	0	0
NEA	−0.007	0.109	−0.009	−0.030	—	0.006	0	0	0	0
GIB	0.226	0.156	0.150	0.184	0.172	—	0.003	0	0	0
MED	0.615	0.430	0.486	0.552	0.524	0.174	—	0	0	0
TAS	0.345	0.234	0.281	0.314	0.306	0.242	0.273	—	0	0
NZ	0.865	0.751	0.844	0.863	0.869	0.798	0.811	0.513	—	0
CL	0.435	0.199	0.337	0.388	0.368	0.301	0.358	0.116	0.399	—
^**φ**^**ST**										
NWA	—	0.002	0.030	0.058	0.145	0	0	0	0	0
FI	0.089	—	0.002	0.001	0.010	0	0	0	0	0
UK	0.043	0.131	—	0.091	0.693	0.001	0	0	0	0
IB	0.029	0.151	0.02	—	0.891	0.001	0	0	0	0
NEA	0.016	0.137	−0.018	−0.014	—	0.001	0	0	0	0
GIB	0.239	0.267	0.218	0.190	0.200	—	0.003	0	0	0
MED	0.578	0.530	0.538	0.540	0.544	0.174	—	0	0	0
TAS	0.253	0.178	0.248	0.260	0.239	0.307	0.394	—	0	0
NZ	0.849	0.762	0.864	0.873	0.878	0.855	0.893	0.421	—	0
CL	0.392	0.219	0.382	0.407	0.378	0.441	0.534	0.086	0.342	—

**Table 4:** Results of the AMOVA among both subspecies of long-finned pilot whales in the South Pacific (*i.e*. *G*. *m*. *edwardii*) and North Atlantic with the Mediterranean (i.e. *G*. *m*. *melas*).

Source of variation	d.f.	Sum of squares	Variance components	Percentage of variation
Among groups	1	124.096	0.23802 Va	39.19
Among populations within groups	8	78.725	0.11293 Vb	18.60
Within populations	1002	256.860	0.25635 Vc	48.33
Total	1011	459.681	0.6073	

**Figure 3:**
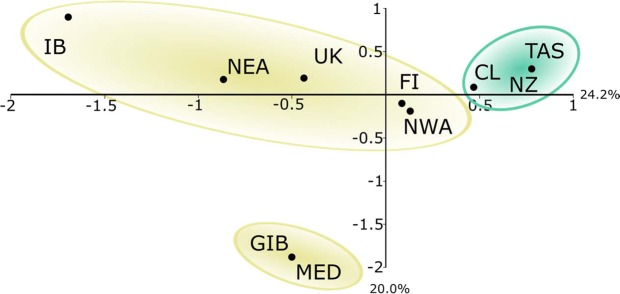
(**a**) Correspondence Analysis scatter plot of presence/absence of haplotypes per locality: Tasmania (TAS), New Zealand (NZ), Chile (CL), northwestern Atlantic (NWA), Faroe Islands (FI), United Kingdom (UK), Iberian Peninsula (IB), northeastern Atlantic (NEA), Strait of Gibraltar (GIB) and the Mediterranean Sea (MED). Localities within the distribution of *G. m. melas* are encircled in yellow and the South Pacific localities of *G. m. edwardii* in green.

In the Correspondence Analysis, the first axis separated the localities according to their respective hemisphere of origin. The three South Pacific localities (TAS, NZ and CL) were clustered separately from those of the Northern Hemisphere. The second axis divided the Strait of Gibraltar and the Mediterranean Sea from the remaining localities in the Northern Hemisphere (Fig. 3). The calculation of Nei’s (1987) net nucleotide divergence (*d*_*A*_) among the subspecies *G. m. edwardii* and *G. m. melas* resulted in *d*_*A*_ = 0.00158 (SD = 0.00019).

### Historical biogeography

The origin of the current disjunct distribution of *Globicephala melas* was explored with DIYABC, using two historical biogeographic models associated to the Last Glacial Maximum (LGM). The first model, scenario 1, represented a colonization event from the Southern Hemisphere to the Northern Hemisphere, followed by isolation and population growth in the Northern Hemisphere. The second model, scenario 2, tested a possible event of vicariance among both distributions.

**Figure 4:**
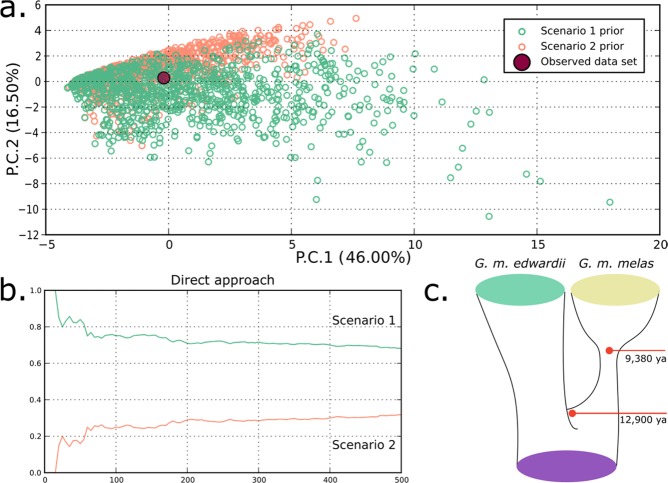
(**a**) PCA showing the scenario and prior combinations of scenario 1 (green) and scenario 2 (orange); (**b**) Estimates of posterior probability of scenarios, via direct approach over the closest 500 scenarios; (**c**) representation of the supported historical biogeographic scenario of colonization.

Based on the genetic data provided, both scenarios were realistic (Fig. 4a), even though the simulations performed were more supportive of the founder effect population history scenario (Fig. 4b). Parameter estimations fell within the proposed ranges and the prior and posterior values from our dataset were not statistically different from simulations. A range expansion would have occurred around 12 900 years ago (t2), followed by a distribution split and population growth, 9 380 years ago (t1) (Fig. 4c). The mutation rate was estimated at u = 4.44 e^*−*8^.

### Microsatellite diversity

Previous data on microsatellite diversity was available for the southwestern Pacific^27^ and the North Atlantic^23,25,28^. The diversity by locus reported^27,28^ is shown in Table 2, with five loci in common to both studies. The number of alleles for each shared locus were, respectively: 409/470 (10 *vs*. 9), 415/416 (9 *vs*. 5), 464/465 (9 *vs*. 6), EV37 (10 *vs*. 6) and EV94 (7 *vs*. 7). Two of these loci were also present in our database of 44 analysed individuals from the southeastern Pacific (464/465 and EV37), with 9 and 15 alleles in total. The upper-tail exact test among the five shared loci of *G. m. edwardii*^27^ and *G. m. melas*^28^ showed that South Pacific samples had significantly more alleles per locus (p = 0.028). By contrast, the upper-tail exact test performed with the 10 loci shared between *G. m. edwardii* from the southwestern^27^ and southeastern Pacific (this study) did not detect a statistically significant difference (p = 0.166).

## Discussion

Sampling cetacean species for population genetic studies has proven to be logistically challenging. A large number of individuals need to be sampled for this kind of studies, but they are generally widespread geographically and low in density. Mass strandings of cetaceans represent unique opportunities to collect large numbers of samples, as in the case of the franciscana *Pontoporia blainvillei*^29^, bottlenose dolphin *Tursiops truncatus*^30^, Cuvier’s beaked whale *Ziphius cavirostris*^31^ and the short-beaked common dolphin *Delphinus delphis*^32^. The long-finned pilot whale is a species for which almost all genetic data has been obtained from sampling mass strandings. A large part of its distribution range has been already sampled in the northwestern and northeastern Atlantic^23–26^, but within their broader distribution in the Southern Hemisphere, only the southwestern Pacific has been covered^20^. This study presents new data from two recent mass standings in southern Chile, in order to produce the first genetic information for *Globicephala melas* in the southeastern Pacific and to integrate this new information into the global phylogeography of the species.

Contrasting tissue quality was found between both strandings, which we attribute to the time of sampling of each event. Our sampling of the Isla Clemente stranding took place an estimated two to three months after occurring^33^, while the Isla Navarino individuals were sampled five days after stranding^34^, precluding tissue the deterioration encountered in the former event. This highlights the importance of swift responses to stranding events, in order to secure the best tissue quality possible.

Prior to conducting mtDNA analyses, we first decided to remove one polymorphic site that exhibited a strong homoplasy signal. This problem in mtDNA sequences of *G. melas* was already detected^20^, but not taken into account for the genetic analyses. Homoplasic sites have been described to hinder the resolution of mtDNA gene trees^35^ and have also been pointed out as potential confounders of evolutionary analysis in the mtDNA control region of humpback whales^36^ and human mtDNA coding regions^37^, among others.

Despite the addition of 90 new sequences from the southeastern Pacific to the set of available sequences in the literature -over 9000 km from New Zealand, the nearest sampled locality- the global haplotype network was expanded by just one private low-frequency haplotype, further confirming the worldwide low mitochondrial diversity of *G. melas*. Such low mtDNA diversity has been reported for other matrilineal odontocetes, including orcas (*π* = 0.52%)^38^, false killer whales (*π* = 0.01–0.30%)^39^ and sperm whales (*π* = 0.131–0.407%)^40^. In contrast, cetaceans with more labile social cohesion such as mysticetes and non-matrilineal odontocetes, generally present much higher nucleotide diversity, a feature that appears to be common among these species^41^. Cultural hitchhiking has been regarded as a driver of their low mitochondrial diversity^41,42^. An analogous effect of behaviour on mitochondrial diversity was described in a resident coastal bottlenose dolphin population in central Chile^43^. This particular population operates akin to pilot whales as it is also composed of adult females and their descendants, both male and female. That study found that the genetic diversity of this matrilineal population (Hd = 0.63; *π* = 0.8%) was lower than that of the non-matrilineal transient-pelagic group adjacent to them (Hd = 0.95; *π* = 1.4%), suggesting the importance of social structure in shaping the pattern of genetic diversity in cetaceans (at least in odontocetes).

As previously reported for other areas^20,23,24,26^, our results show that southeastern Pacific long-finned pilot whales (n = 90) have low genetic diversity, in particular for haplotype richness (h = 4) and nucleotide diversity (*π* = 0.23%, Table 1). Within the South Pacific, the diversity indices of southeastern Pacific (*i.e*. Chilean) samples were similar to the values obtained for Tasmania. Nevertheless, genetic diversity in these two localities was very different from that of New Zealand. Despite accounting for 54% of the samples in this basin, the genetic and nucleotide diversity of pilot whales from New Zealand were the lowest (Table 1), particularly because of the high predominance of one haplotype (93%, haplotype P + U). Such overrepresentation of a single haplotype in a matrilineal species may derive from sampling bias, for example, by sampling a single, large mass stranding event. However, in that study, the sampling period spanned from 1993 to 2007 and included numerous single and mass strandings that took place in various localities^20^. Therefore, we can assume that this diversity accurately reflects what is present in this area. The distinctiveness of New Zealand from Tasmania and Chile is further supported by high *φ*_*ST*_ values (*φ*_*ST*_ = 0.421 and 0.342 respectively) (Table 3), which are four and five times higher than between Chile and Tasmania (*φ*_*ST*_ = 0.086). The absence of obvious geographic or oceanographic barriers between New Zealand and the other localities in the South Pacific does not allow a simple interpretation of the pattern of genetic structure found here. This genetic differentiation could have been attained through ecological specialization, as previously pointed out^20^. Similarly, sea surface temperature and its influence on prey distribution has been regarded as a possible ecological factor underlying genetic differentiation in extant *G. melas* in North Atlantic waters^28^ and similar trends have been observed on the Scotian shelf^44^.

Differentiation over relatively short distances without any conspicuous geographical barriers has also been detected in other odontocetes. For example, genetic differentiation was detected in the Chilean dolphin *Cephalorhynchus eutropia* between two differing coastal habitats along the uninterrupted Chilean coastline, attributed to habitat adaptation and specific hunting strategies^45^. Also, the Eastern Pacific Barrier has been proposed as a driver behind the genetic differentiation of the short-finned pilot whale *Globicephala macrorhynchus* into two subspecies^46^.

**Figure 5:**
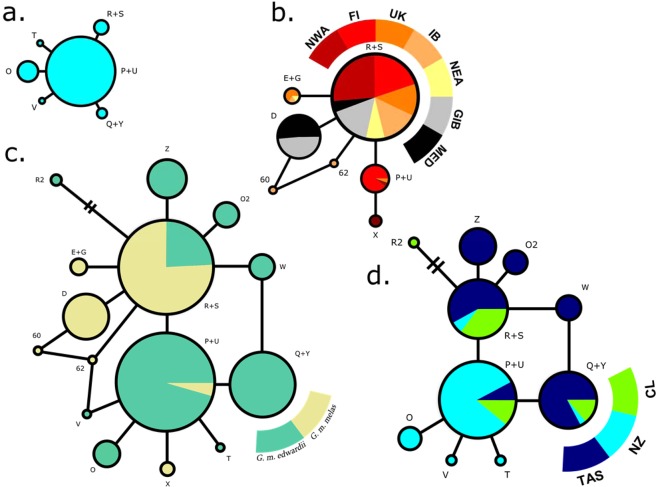
Haplotype networks of (**a**) New Zealand samples, (**b**) North Atlantic and Mediterranean samples, (**c**) Global dataset coloured by subspecies and (**d**) South Pacific samples. Smallest circle size indicates a frequency of one sequence. Included localities are Tasmania (TAS), New Zealand (NZ), Chile (CL), northwestern Atlantic (NWA), Faroe Islands (FI), United Kingdom (UK), Iberian Peninsula (IB), northeastern Atlantic (NEA), Strait of Gibraltar (GIB) and the Mediterranean Sea (MED). Detailed haplotype frequencies can be found in Table 1.

The haplotype network of the specimens from New Zealand presents a typical star-like shape (Fig. 5a), suggesting that long-finned pilot whales around New Zealand represent a young population, perhaps tracing back to the Last Glacial Maximum, as suggested by DIYABC analyses. During this period, a 6–10 °C cooling occurred in superficial waters of southeast New Zealand, the strongest temperature drop reported in this area of the southwestern Pacific^47^. Such changes in environmental conditions, probably associated to a shift in the distribution of marine biota, may have provoked a typical population contraction-expansion in the long-finned pilot whale population in this area, as described for cold-temperate and polar marine species^48^, including cetaceans^49,50^.

A similar case of strong genetic differentiation among populations of long-finned pilot whales is observed between the North Atlantic and Mediterranean populations, where the latter exhibits high phylogeographic and genetic differences with the North Atlantic localities^25^. In this case, geographic and oceanographic discontinuities between the Mediterranean Sea and the Atlantic Ocean provide a robust explanation for the observed genetic structure. The separation of Mediterranean populations from North Atlantic ones has been previously reported in various marine species, such as shallow water crustaceans^51^, sea stars^52^, white sharks^53^ and other odontocetes, like in sperm whales^54^, striped dolphins *Stenella coeruleoalba*^55^ as well as Cuvier’s beaked whales *Ziphius cavirostris*^31^ and Risso’s dolphins *Grampus griseus*^56^.

Widespread cetacean taxa occurring in both the Northern and Southern Hemispheres generally exhibit strong phylogeographic structure and genetic divergence between regions. Such genetic differentiation has been generally exemplified by fixed substitutions in mtDNA control region sequences. This is the case of the fin whale *Balaenoptera physalus*, with one fixed difference between South and North Atlantic samples, thus presenting no shared haplotypes^57^, the harbour porpoise *Phocoena phocoena*, with high divergence among ocean basins^58^ and the more closely related false killer whale *Pseudorca crassidens*^39^. In the latter species, although the study had a low sample size in the North Atlantic, no shared haplotypes were found and at least 10 substitutions separated the Atlantic populations from those in the Indo-Pacific. In the case of *G. melas*, despite its antitropical distribution and the large geographic discontinuity between northern and southern distribution areas, the subspecies shared their two most abundant haplotypes. This genetic pattern may reflect (1) contemporary gene flow between hemispheres, or alternatively (2) an ancestral polymorphism resulting from an incipient divergence process. The strong phylogeographic structure detected among the subspecies supports the second hypothesis.

The South Pacific network holds much of the species’ genetic diversity and is therefore very similar to the global network in overall shape (Fig. 5b). In contrast, the star-like haplotype network of North Atlantic and Mediterranean samples is typically presented by recently expanding populations. A biogeographic scenario of a dispersal event over the equator during previous glacial period has been proposed^22^, with a split in distribution occurred after the Last Glacial Maximum, 10 000–15 000 years ago. This hypothesis was further expanded^20^, mentioning that a trans-equatorial dispersal event rather than vicariance might have taken place, on account of the lower diversity in the Northern Hemisphere subspecies. Thus, the hypothesis is supported by (1) the contrasting haplotype networks (Fig. 5c, d), (2) the higher mitochondrial and microsatellite diversity indices of South Pacific long-finned pilot whales compared to their counterparts in the North Atlantic and Mediterranean (Tables 1 and 2) and (3) the clear separation of areas by hemisphere in the haplotype presence/absence Correspondence Analysis (Fig. 3). The validity of the previously proposed scenario of population history was further explored here with DIYABC population history simulations, comparing a founder effect scenario with a vicariance scenario. The patterns of population genetic diversity and structure observed in *G. melas* were consistent with the data simulated under the former scenario, a trans-equatorial dispersal event followed by divergence (Fig. 4). Posterior estimation of scenario parameters allowed estimating the time at which the different events occurred, setting the dispersal from the Southern Hemisphere to the North Atlantic at around 13 000 years ago, followed by a population demographic expansion around 9 380 years ago. However, even considering this scenario of divergence, the absence of reciprocal monophyly does not qualify northern and southern *G. melas* as different Evolutionary Significant Units (ESU) following the criterion of Moritz (1994)^21^. It is likely that not enough time has passed to sort lineages, or some level of gene flow still occurs^59^, as they still share haplotypes, but in differing frequencies^20^. Additionally, net nucleotide divergence *d*_*A*_ and Percent Diagnosability (PD) had been previously calculated for the long-finned pilot whale subspecies^60^, obtaining values of *d*_*A*_ = 0.00128 and PD = 0.84286. Our calculation of net nucleotide divergence, which included new sequences from the southeastern Pacific and integrated other published sequences from the Northern Hemisphere, resulted in a slightly higher value of *d*_*A*_ = 0.00158, which still is below the proposed subspecies interval^61^. Therefore, addressing them as *Demographically Independent Populations* (DIP), a transitional state between a panmictic population and separate ESUs^59^ might be more accurate.

Finally, from a conservation perspective, even if genetic analyses do not support the subspecies category, we recommend maintaining their current taxonomic status, since these DIP might be undergoing a recent divergence process which has yet to mature into fully sorted lineages.

## Concluding Remarks

The results here presented should be considered as preliminary evidence, as the use of a single mtDNA marker for phylogeographic and demographic inferences has been deemed problematic before^62^. As previously stated, such molecular studies should include nuclear markers together with mitochondrial DNA, even more when delimitating species and subspecies^18^. However, to date, the mtDNA control region is the only molecular marker for which sequences are available with sufficient sample size from different ocean basins to perform a worldwide phylogeographic study in *G. melas*^26^. To incorporate new genetic markers to a global phylogeographic study would require tremendous sampling effort and expenses, and may also strongly depend on the occurrence of mass strandings in all these regions of the world.

Finally, we believe that collaborative studies surveying uncharted areas, especially within the greater distribution range of long-finned pilot whales in the Southern Hemisphere, are fundamental to obtain complete data on the worldwide phylogeography and taxonomy of *G. melas* and to lay the groundwork for future research on these topics. Modern genetic tools, such as complete mitogenome and SNPs, have been already used to revise the taxonomic status of short-finned pilot whales^46^, which could be replicated in long-finned pilot whales.

## Methods

### Southeastern pacific sample collection

Tissue samples were collected from twelve individuals in a stranding event that occurred in Isla Navarino in 2006 (55°15′S; 67°30′W (Fig. 1). In 2016, 124 *Globicephala melas* were sampled from the mass stranding event that occurred in Isla Clemente 45°35′57.50″S; 74°34′30.32″ W. All samples were preserved in 90–95% ethanol.

### DNA extraction, mitochondrial control region sequencing and microsatellite genotyping

DNA extractions were performed following a modified salt-extraction protocol^63^, adding a second step of digestion with proteinase K one hour after the first one.

### Mitochondrial control region data

The mtDNA control region was amplified using the primers M13 Dlp1.5 5′-TGTAAAACGACAGCCAGTTCACCCAAAGCTG RARTTCTA-3′ (forward) and 8 G 5′-GGAGTACTATGTCCTGTAACCA-3′ (reverse)^31^. The amplification protocol was as follows: 25.6 *µ*L reaction volume for each PCR reaction consisted of 12.7 *µ*L water, 5 *µ*L 10X Buffer (Invitrogen), 2 *µ*L 50 mM MgCl_2_ (Invitrogen), 2 *µ*L 10pM dNTPs (Invitrogen), 1 *µ*L 10pM of each primer (2 *µ*L total), 0.5 *µ*L Taq polymerase (Invitrogen) and 70–150 ng of DNA. A Thermo Hybaid PxE 0.5 thermal cycler was used for all amplifications, with the following profile: Preliminary denaturation of 2 minutes at 94 °C; followed by 30 cycles of: denaturation for 30 s at 94 °C, annealing for 40 s at 56 °C, polymerase extension for 40 s at 72 °C; and a final polymerase extension for 10 minutes at 72 °C and an infinite hold temperature of 4 °C. Each PCR run included positive and negative controls. Fragments were run in a 1% agarose gel, each well containing 3 *µ*L of PCR product mixed with an equal volume of loading dye with 0.3% Gel Red and visualized in a gel documentation system (Maestrogen SMU-01).

PCR product purification and sequencing in both directions were done at Macrogen Inc., Seoul, South Korea with a 3730XL DNA Analyzer (Applied Biosystems). All sequences obtained were aligned manually in ProSeq 3.5^64^ and polymorphic sites were visually checked. Prior to molecular analyses, the species for each sample was corroborated with two platforms of comparative analysis of sequences: BLAST (Basic Local Alignment Search Tool, www.blast.ncbi.nlm.nih.gov) and DNA Surveillance^65^.

An additional 922 control region sequences were obtained from five other sources: (1) Oremus *et al*., (2009)^20^ (n = 573, Tasmania and New Zealand, GenBank access codes: FJ513342-54); (2) Siemann (1994)^26^ (n = 59; western North Atlantic, Cape Cod, Newfoundland, Nova Scotia, Scotland and England; GenBank access codes: U20926-28); (3) Monteiro (2013)^24^ (n = 116, eastern coast of the United States, Faroe Islands, United Kingdom and Iberian Peninsula, GenBank access codes: KC934932-34); (4) Verborgh (2015)^25^ (n = 117, eastern coast of the United States, Faroe Islands, United Kingdom, Euskadi, northeastern Atlantic, Iberian Peninsula, Strait of Gibraltar and the Mediterranean Sea; haplotypes reconstructed by hand) and (5) Miralles *et al*., (2016)^23^ (n = 57, Faroe Islands and Iberian Peninsula, GenBank access codes: KJ740360-71) (Table 1).

### Sample grouping

Samples were grouped in two ways: (1) first in ten groups, according to their respective sampling locality: Tasmania (TAS), New Zealand (NZ), Chile (CL), northwestern Atlantic (NWA), Faroe Islands (FI), United Kingdom (UK), Iberian Peninsula (IB), northeastern Atlantic (NEA), Strait of Gibraltar (GIB) and the Mediterranean Sea (MED). NEA included samples ranging from UK to IB, and was combined with the locality Euskadi from the original study, since no significant *F*_*ST*_ structure was detected among them in that study^25^ (Fig. 1, Table 1). (2) The second way of grouping samples was according to the subspecies categories, *i.e. G. m. edwardii* from the South Pacific (SP) including the localities TAS, NZ and CL and *G. m. melas* from the North Atlantic (NA), including NWA, FI, UK, IB, NEA and the Mediterranean (GIB and MED).

### Sequence editing

After alignment and trimming, a haplotype network was constructed in Network 5.0^66^. With an exploratory examination of the global haplotype network, it was noted that site 156 of the alignment generated three loops in the network. This hypervariable site was considered to be interfering with the phylogeographic signal of the data and was consequently removed, in order to eliminate a potential homoplasy signal. Additionally, a repeated TA motif starting at position 90 was identified as a possible microsatellite. We modified the sequences by deleting one of the nucleotide positions within each repeat, so each motif was considered as a single mutational step, instead of each nucleotide separately. Thus the final fragment length was of 345 bp.

### Genetic diversity and structure

The genetic diversity indices number of haplotypes (h), number of polymorphic sites (S), haplotype diversity (Hd), nucleotide diversity (*π*) and pairwise differences between sequences (Π) were estimated in Arlequin v3.5.2^67^. Analyses of genetic structure (*F*_*ST*_), phylogeographic structure (*φ*_*ST*_) and analysis of molecular variance (AMOVA) were conducted in Arlequin v3.5.2 with 1000 permutations and a significance level of 0.05. Phylogeographic structure was also explored with Snn tests of genetic differentiation^68^, performed in DnaSP 5.10.01^69^. For the AMOVA, the ten localities were grouped according to the distribution of each subspecies. A Correspondence Analysis (CA) was performed on all localities with the software Past 3.19^70^, using the matrix of Table 1 in the form of presence/absence of haplotypes.

Additionally, as suggested by the guidelines for the delimitation of cetacean subspecies using genetic data of Taylor *et al*.^61^, Nei’s (1987) net nucleotide divergence (*d*_*A*_, equation 10.21)^71^ was calculated among the two putative subspecies in DnaSP. According to these guidelines, the net nucleotide divergence among two subspecies should be within the range of *d*_*A*_ = 0.004–0.04.

### Historical biogeography

**Figure 6:**
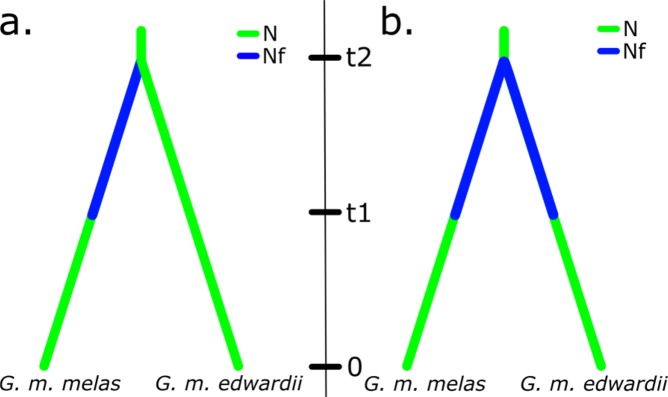
Proposed historical biogeographic scenarios tested in DIYABC: (**a**) trans-equatorial colonization event followed by divergence and (**b**) vicariance event. N = Previous effective population size, *N*_*f*_ = founder effect effective population size. Time scale: 0 = present, *t*_1_ = population size expansion, *t*_2_ = a. colonization event or b. population split.

The population history of the species was tested on the program DIYABC v2.1.0^72^. This software evaluates population histories using Approximate Bayesian Computation (ABC) with genetic data, by testing scenarios built through the combination of population divergence, admixture and population size changes. Two models were evaluated. The first model was defined based on a scenario previously proposed^22^, together with evidence from the genetic results provided in the present study. The model considers a trans-equatorial, Last Glacial Maximum (LGM)-associated dispersal event from the Southern Hemisphere to the Northern Hemisphere, followed by a split in distribution and instantaneous population growth (Fig. 6). The alternative model differs from the previous one in that it considers a vicariance event, rather than a founder effect. The program was used to evaluate the accordance of these two scenarios with our genetic data. Priors were set as follows: Effective population size (*N*_*e*_) of ancient population = 1 000–100 000; Effective population size of founder effect (*N*_*f*_) = 10–1 000; time of dispersal event *t*_2_ = 10 000–35 000; time of instantaneous population growth *t*_1_ = 2 000–15 000 (with *t*_2_ > *t*_1_) and mutation rate u = 1.5 e^*−*7^–1.5 e^*−*8^. In accordance with the recommendations of the authors of the software, we performed 6 000 000 simulations.

### Microsatellite data

A total of 19 loci were amplified: 409/470, 464/465^73^, DlrFCB1, DlrFCB6^74^, Ev1, Ev14, EV37^75^, GATA53^76^, GT6, GT51^77^, GT23, GT211, GT509, GT575^78^, MK5, MK9^79^ and PPHO131^58^. PCR reactions were done with a Multiplex PCR kit (Qiagen), each reaction containing: 12.5 *µ*L of water, 5 *µ*L of MM2x Buffer and 1 *µ*L of each primer at 10 pM. Between two and four loci with different fluorescent dyes were combined in each reaction. Allele scoring was done with the software GeneMarker v2.6.0 (www.softgenetics.com) with a 500 liz standard. The dataset was tested for scoring errors, allele dropout and null alleles in Micro-Checker v.2.2.3^80^. Observed heterozygosity (*H*_*o*_), expected heterozygosity (*H*_*e*_), average number of alleles per locus (nA) and genetic structure (*F*_*ST*_) were estimated in Genetix v4.05.2^81^. Published microsatellite data on this species was available from Tasmania and New Zealand^27^, NWA, UK^28^, Faroe Islands^23,28^, Iberian Peninsula^23^, northeastern Atlantic, Strait of Gibraltar and the Mediterranean^25^. However, because of the differences in the loci used in these studies, only a partial comparison of genetic diversity could be performed between populations of *G. m. melas* and *G. m. edwardii*. Only comparisons of allelic richness could be done, which were carried out in Rundom Pro 1.1^82^ with 10 000 randomizations. Comparisons were intra-subspecies among southwestern and southeastern Pacific samples, and inter-subspecies among southwestern Pacific and North Atlantic samples.

### Approval

We confirm that all methods were carried out in accordance with relevant guidelines and regulations. Samples were taken from stranded, deceased animals with permission from the National Fisheries Service (SERNAPESCA, document ID 2016-11-13). All experimental protocols were approved by the Postgraduate Evaluation Committee at the Faculty of Science of the Universidad de Chile.

### Accession codes

Haplotype R2 (GenBank accession number pending. Submission number #2305260).
